# Evolution of microgastropods (Ellobioidea, Carychiidae): integrating taxonomic, phylogenetic and evolutionary hypotheses

**DOI:** 10.1186/1471-2148-13-18

**Published:** 2013-01-23

**Authors:** Alexander M Weigand, Adrienne Jochum, Rajko Slapnik, Jan Schnitzler, Eugenia Zarza, Annette Klussmann-Kolb

**Affiliations:** 1 Department of Phylogeny and Systematics, Institute for Ecology, Evolution and Diversity, Biosciences, Goethe-University Frankfurt, Max-von-Laue Straße 13, 60438, Frankfurt am Main, Germany; 2 Institute of Biology, Center for Scientific Research of the Slovenian Academy of Sciences and Arts, Novi trg 2, p.p. 306, 1000, Ljubljana, Slovenia; 3 Biodiversity and Climate Research Centre, Senckenberganlage 25, 60325, Frankfurt am Main, Germany

**Keywords:** Integrative taxonomy, Protracted speciation, Subterranean environment, Cryptic diversity, DNA barcoding, Allopatric diversification, *Carychium*, *Zospeum*, Phylogeny, Gastropoda

## Abstract

**Background:**

Current biodiversity patterns are considered largely the result of past climatic and tectonic changes. In an integrative approach, we combine taxonomic and phylogenetic hypotheses to analyze temporal and geographic diversification of epigean (*Carychium*) and subterranean (*Zospeum*) evolutionary lineages in Carychiidae (Eupulmonata, Ellobioidea). We explicitly test three hypotheses: 1) morphospecies encompass unrecognized evolutionary lineages, 2) limited dispersal results in a close genetic relationship of geographical proximally distributed taxa and 3) major climatic and tectonic events had an impact on lineage diversification within Carychiidae.

**Results:**

Initial morphospecies assignments were investigated by different molecular delimitation approaches (threshold, ABGD, GMYC and SP). Despite a conservative delimitation strategy, carychiid morphospecies comprise a great number of unrecognized evolutionary lineages. We attribute this phenomenon to historic underestimation of morphological stasis and phenotypic variability amongst lineages. The first molecular phylogenetic hypothesis for the Carychiidae (based on COI, 16S and H3) reveals *Carychium* and *Zospeum* to be reciprocally monophyletic. Geographical proximally distributed lineages are often closely related. The temporal diversification of Carychiidae is best described by a constant rate model of diversification. The evolution of Carychiidae is characterized by relatively few (long distance) colonization events. We find support for an Asian origin of *Carychium*. *Zospeum* may have arrived in Europe before extant members of *Carychium*. Distantly related *Carychium* clades inhabit a wide spectrum of the available bioclimatic niche and demonstrate considerable niche overlap.

**Conclusions:**

Carychiid taxonomy is in dire need of revision. An inferred wide distribution and variable phenotype suggest underestimated diversity in *Zospeum*. Several *Carychium* morphospecies are results of past taxonomic lumping. By collecting populations at their type locality, molecular investigations are able to link historic morphospecies assignments to their respective evolutionary lineage. We propose that rare founder populations initially colonized a continent or cave system. Subsequent passive dispersal into adjacent areas led to *in situ* pan-continental or mountain range diversifications. Major environmental changes did not influence carychiid diversification. However, certain molecular delimitation methods indicated a recent decrease in diversification rate. We attribute this decrease to protracted speciation.

## Background

Climatic and geological changes are considered to be major drivers of biological diversification. Many well-characterized radiations were initiated in aftermath of major geologic events [1-3]. Current biodiversity patterns reflect these consequential processes. While taxa with low dispersal ability may be particularly sensitive to changes in their environment, historically-formed patterns within these taxa are known to remain well preserved [4,5]. The taxon Ellobioidea (Gastropoda, Eupulmonata) comprises a group of morphologically and ecologically highly diverse snails, known to have successfully invaded the marine, brackish water and terrestrial habitats [6,7]. Species are traditionally classified into five taxonomic groups, the Pythiidae, the Laemodontidae, the Melampodidae, the Ellobiidae and the Carychiidae. These taxa have been recognized either as families within Ellobioidea or as sub-families within the family Ellobiidae [6-8]. To avoid confusion, we will here refer to the taxon Ellobioidea and to families. Taxonomic descriptions and systematic classifications were exclusively based on morphological (anatomical and conchological) characters while extant species show a mosaic pattern of plesiomorphic and apomorphic features resulting from convergent evolution [6,7,9,10]. Due to the tenuous nature of morphological characters, phylogenetic reconstructions are extremely difficult [6,11]. Moreover, the high degree of homoplasy in morphological characters and frequent low variability has led to the description of approximately 800 species names available in the literature, whereby 250 are likely to be valid [12]. The most comprehensive molecular study for the Ellobioidea suggests a monophyletic origin of the entire group. However, the relationships among the five traditional ellobioid taxa are still unclear [13].

One lineage of the Ellobioidea, the Carychiidae Jeffreys, 1830 has successfully accomplished a complete transition onto land. Extant carychiid snails inhabit aphotic and permanently wet epigean (*Carychium*) or subterranean (*Zospeum*) environments throughout their Holarctic distribution. This dramatic shift from a marine to a terrestrial habitat has occurred independently of the stylommatophoran land-snails of the Eupulmonata [13,14]. As for all Ellobioidea, taxonomic and systematic descriptions of Carychiidae are based upon characters of the mature shell, which in the case of carychiid gastropods, are suspected to vary according to environmental conditions [15-17]. The first attempt to characterize carychiid taxa using DNA barcodes supported 90% of traditional morphospecies assignments [18]. Nevertheless, the same study only addressed a single population or a few populations per morphospecies. Phenotypic variability (in *Carychium*) and morphological stasis (in *Zospeum*) were identified as potential explanations for discrepancies in morphological and molecular taxonomy. In particular, *Zospeum* displayed high intraspecific genetic diversity within single morphospecies, as reflected by several cave-endemic evolutionary lineages (ELs). In the case of European *Carychium*, DNA barcoding revealed a previously overlooked taxonomic entity and helped to reevaluate the taxonomic status of questionable morphospecies [18,19]. Additionally, the phenotypically variable *Carychium* shell was shown to span a wide range of shell dimension and proportions, encompassing three morphospecies. Hence, it is very likely that the Carychiidae harbor a considerable number of morphologically unrecognized ELs.

A gastropod’s migratory ability is correlated with its shell size [20,21]. Due to their small size, the active dispersal abilities of carychiid snails are highly limited. Slapnik [22] conducted investigations on *Zospeum isselianum* activity within a cave showing moving distances measuring 1 to 15 cm per week (on average 0.7 cm per day). In a comparative phylogeography of two European *Carychium* species, Weigand et al. [23] revealed that the local population structure is often formed by only a few mtDNA haplotypes suggesting that adjacent populations may only exhibit infrequent gene flow. Nevertheless, minute gastropods are well adapted to passive dispersal (e.g. [24,25]), a mechanism that best explains the foundation of transatlantic European *Carychium* populations in North America [26,27] and the postglacial recolonization of Northern Europe [23]. Although passive dispersal events are relatively rare, these, along with limited mobility, could well contribute to a higher incidence of isolated populations and narrow endemism.

Here, we use an integrative approach combining taxonomic, phylogenetic and evolutionary hypotheses to assess the diversification of ELs in Carychiidae. This process first assigned all specimens to phenotype hypotheses. A conservative genetic delimitation method is used to evaluate the initial morphospecies assignments and to identify distinct ELs [28,29]. This approach allows us to include otherwise morphologically unrecognized ELs, whose absence during phylogenetic tree reconstructions could yield misleading results [30]. Based on the identified lineages, a molecular phylogenetic hypothesis for Carychiidae is then reconstructed to test three evolutionary hypotheses: 1) morphospecies encompass unrecognized ELs, 2) limited dispersal results in a close genetic relationship of geographical proximally distributed taxa and 3) major climatic and tectonic events had an impact on lineage diversification within Carychiidae.

## Results

### Molecular identification of evolutionary lineages and species delimitation

**Figure 1:**
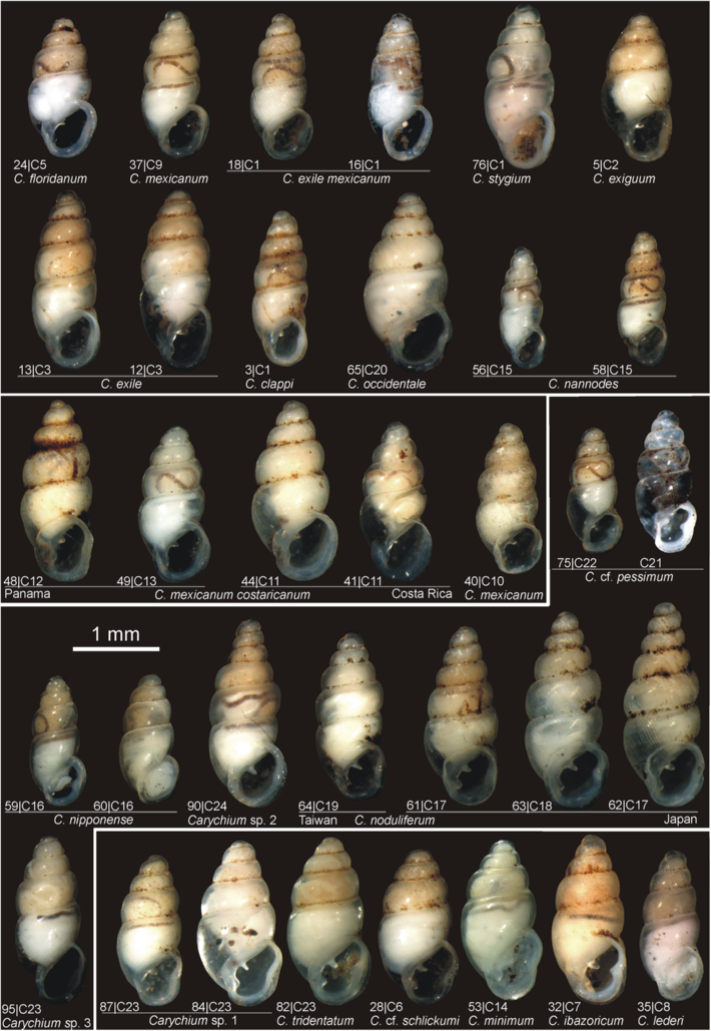
***Carychium *morphospecies and corresponding genetic lineages.** Traditionally identified morphospecies and their respective delimitated evolutionary lineage (C1-C25) and specimen identifier are visualized. The lineage C21 (*C.* cf. *pessimum*) is figured by an empty shell of the same population instead of an analyzed individual.

**Figure 2:**
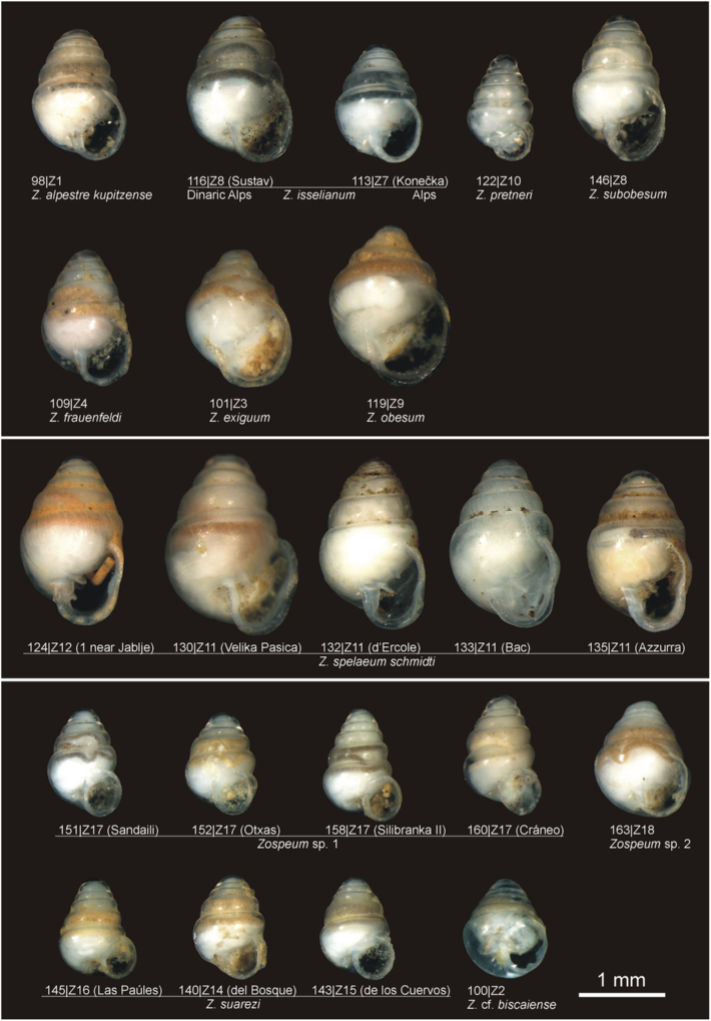
***Zospeum *morphospecies and corresponding genetic lineages.** Traditionally identified morphospecies and their respective delimitated evolutionary lineage (Z1-Z18) and specimen identifier are visualized. In some cases the cave name is provided in brackets.

**Figure 3:**
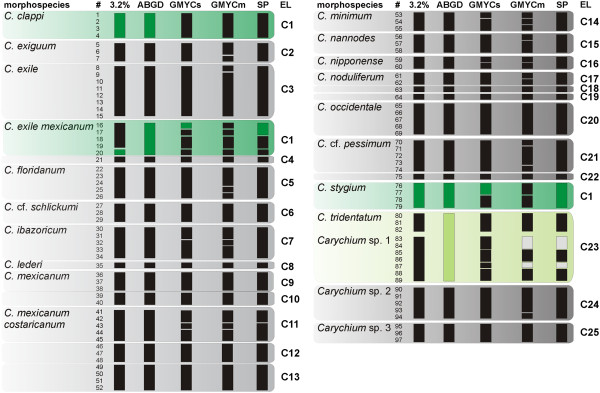
**Molecular delimitation of *Carychium *evolutionary lineages.** Results of the five genetic delimitation approaches are indicated. For *Carychium*, 97 specimens (#) comprising 18 morphospecies and three undescribed taxa are analysed. A conservative strategy identified 25 evolutionary lineages (EL; C1-C25). Specimens clusters identified under each single delimitation method (3.2%, ABGD, GMYCs, GMYCm and SP) are indicated by black boxes. Grey boxes refer to clustered specimens within a single morphospecies (e.g. SP, # ‘83’ + ‘84’ + ‘87’), colored boxes to specimens between different morphospecies (e.g. SP, # ‘16’ + ‘17’ + ‘76-79’).

**Figure 4:**
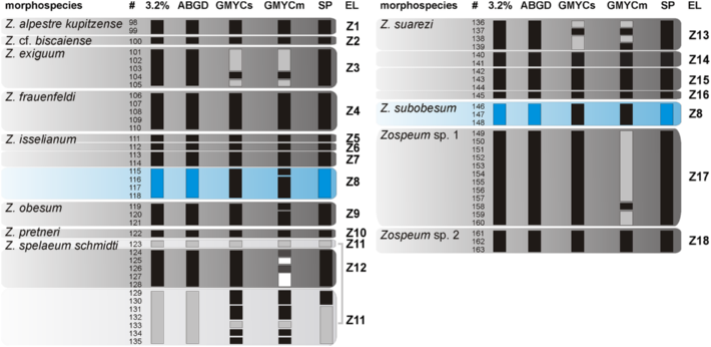
**Molecular delimitation of *Zospeum *evolutionary lineages.** Results of the five genetic delimitation approaches are indicated. For *Zospeum*, 66 specimens (#) comprising 10 morphospecies and two undescribed taxa are analysed. A conservative strategy identified 18 evolutionary lineages (EL; Z1-Z18). Specimens clusters identified under each single delimitation method (3.2%, ABGD, GMYCs, GMYCm and SP) are indicated by black boxes. Grey boxes refer to clustered specimens within a single morphospecies (e.g. 3.2%, # ‘123’ + ‘129-135’), colored boxes to specimens between different morphospecies (e.g. 3.2%, # ‘115-118’ + ‘146-148’).

To address morphologically unrecognized ELs, the initial morphospecies identifications (Figures 1 and 2, Table 1) were examined applying a combination of five molecular delimitation approaches on mitochondrial sequence data. The 3.2% K2P threshold value identified 45 partitions (Figures 3 and 4). The Automatic Barcoding Gap Detection (ABGD) method consistently revealed 43 ELs for all tested combinations. Two versions of the General Mixed Yule-Coalescent (GMYC) model were applied. Both variants performed significantly better than the null model of a single coalescent population (L_0_ = 1275.858, both p-values < 0.0001), but led to to a high partitioning of the dataset resulting in 64 (L_GMYCs_ = 1315.46) and 78 clusters (L_GMYCm_ = 1321.515), respectively. No significant improvement was found when applying GMYCm instead of GMYCs (Chi-square = 12.1081, df = 6 and p = 0.0596). Finally, the Statistical Parsimony (SP) approach delimited 51 ELs mostly congruent to the 3.2% threshold and ABGD results.

**Table 1:** Individuals and sampling localities

**morphospecies**	**#**	**locality**	**geographical position**		**EL**
***Carychium*** O.F. Müller, 1774			**latitude**	**longitude**	
*C. clappi*	1	USA, Tennessee, La Follette	36.332	−83.998	C1
Hubricht, 1959	2	USA, Tennessee, La Follette	36.332	−83.998	C1
	3	USA, Tennessee, La Follette	36.332	−83.998	C1
	4	USA, Tennessee, La Follette	36.332	−83.998	C1
*C. exiguum*	5	USA, New York, Naples, Ontario County, Grimes Glen	42.61591	−77.41355	C2
Say, 1822	6	USA, New York, Naples, Ontario County, Grimes Glen	42.61591	−77.41355	C2
	7	USA, New York, Naples, Ontario County, Grimes Glen	42.61591	−77.41355	C2
*C. exile*	8	USA, Indiana, Lawrence County, Williams Cave Sinkhole	38.7555	−86.59105	C3
H. C. Lea, 1842	9	USA, Indiana, Lawrence County, Williams Cave Sinkhole	38.7555	−86.59105	C3
	10	USA, Indiana, Lawrence County, Williams Cave Sinkhole	38.7555	−86.59105	C3
	11	USA, New York, Portageville, Letchworth State Park	42.57909	−78.04945	C3
	12	USA, New York, Portageville, Letchworth State Park	42.57909	−78.04945	C3
	13	USA, New York, Watkins Glen	42.375828	−76.871115	C3
	14	USA, New York, Watkins Glen	42.375828	−76.871115	C3
	15	USA, New York, Watkins Glen	42.375828	−76.871115	C3
*C. exile mexicanum*	16	USA, Georgia, Adairsville, Bartow County, Barnsley Gardens	34.311233	−84.9866	C1
Pilsbry, 1891	17	USA, Georgia, Adairsville, Bartow County, Barnsley Gardens	34.311233	−84.9866	C1
	18	USA, Alabama, Little River Mouth Park, Cherokee County	34.312233	−85.685733	C1
	19	USA, Alabama, Little River Mouth Park, Cherokee County	34.312233	−85.685733	C1
	20	USA, Illinois, Jackson County, Gorham	37.6865	−89.490667	C1
	21	USA, Florida, Marianna	30.810556	−85.226667	C4
*C. floridanum*	22	USA, Florida, Wakulla Springs	30.23548	−84.303087	C5
Clapp, 1918	23	USA, Florida, Wakulla Springs	30.23548	−84.303087	C5
	24	USA, Florida, Wakulla Springs	30.23548	−84.303087	C5
	25	USA, Florida, Wakulla Springs	30.23548	−84.303087	C5
	26	USA, Florida, Wakulla Springs	30.23548	−84.303087	C5
*C.* cf. *schlickumi*	27	Greece, Epirus, Ioáninna Prov., Métsovo	39.8213	21.1294	C6
Strauch, 1977	28	Greece, Epirus, Ioáninna Prov., Métsovo	39.8213	21.1294	C6
	29	Greece, Epirus, Ioáninna Prov., Métsovo	39.8213	21.1294	C6
*C. ibazoricum*	30	Portugal, Azores, San Miguel, Sete Cidades	37.847033	−25.780217	C7
Bank & Gittenberger, 1985	31	Portugal, Azores, San Miguel, Furnas	37.770383	−25.306583	C7
	32	Portugal, Azores, San Miguel, Furnas	37.770383	−25.306583	C7
	33	Portugal, Estremadura, near Sao Pedro de Moel	39.774167	−9.016667	C7
	34	Portugal, Estremadura, near Sao Pedro de Moel	39.774167	−9.016667	C7
*C. lederi* O. Boettger, 1880	35	Iran, Mazandaran, Nowshahr, Kheiroudkanar forest	36.605833	51.568333	C8
*C. mexicanum*	36	USA, Georgia, Flovilla, Butts County, Indian Springs State Park	33.242367	−83.92035	C9
Pilsbry, 1891	37	USA, Georgia, Flovilla, Butts County, Indian Springs State Park	33.242367	−83.92035	C9
	38	USA, Georgia, Flovilla, Butts County, Indian Springs State Park	33.242367	−83.92035	C9
	39	Belize, Maya Mountains, Bladen Nature Reserve	16.557167	−88.707833	C10
	40	Belize, Maya Mountains, Bladen Nature Reserve	16.557167	−88.707833	C10
*C. mexicanum costaricanum*	41	Costa Rica, Puntarenas, Santa Elena	10.369833	−84.804167	C11
Von Martens, 1898	42	Costa Rica, Puntarenas, Monte Verde	10.301333	−84.790167	C11
	43	Costa Rica, San José, San Gerardo de Dota	9.5496	−83.8032	C11
	44	Costa Rica, San José, San Gerardo de Dota	9.5496	−83.8032	C11
	45	Costa Rica, San José, San Gerardo de Dota	9.5496	−83.8032	C11
	46	Panama, Chiriquí, Sendero el Retoño, Parque internacional la Amistad	8.8913	−82.628611	C12
	47	Panama, Chiriquí, Sendero el Retoño, Parque internacional la Amistad	8.88815	−82.620667	C12
	48	Panama, Chiriquí, Sendero el Retoño, Parque internacional la Amistad	8.88815	−82.620667	C12
	49	Panama, Chiriquí, near Boquete	8.824767	−82.495833	C13
	50	Panama, Chiriquí, near Boquete	8.824767	−82.495833	C13
	51	Panama, Chiriquí, near Boquete	8.824767	−82.495833	C13
	52	Panama, Chiriquí, near Boquete	8.824767	−82.495833	C13
*C. minimum*	53	Poland, Słubice	52.3467	14.5806	C14
O.F. Müller, 1774	54	Italy, Upper Adige, near Auer	46.3385	11.3503	C14
	55	Spain, Asturias, Gijón	43.5204	−5.6164	C14
*C. nannodes*	56	USA, Tennessee, Overton County, Slit Cave	36.456833	−85.375067	C15
G.H. Clapp, 1905	57	USA, Tennessee, Unicoi County, near Unaka Springs	36.0986	−82.4466	C15
	58	Canada, Ontario, Crawford Lake	43.4712	−79.9464	C15
*C. nipponense*	59	Japan, Honshu Is., Iwate, Ichinoseki, Geibikei	38.9845	141.255667	C16
Pilsbry & Hirase, 1904	60	Japan, Honshu Is., Fukushima, Urabandai	37.687783	140.1205	C16
*C. noduliferum*	61	Japan, Honshu Is., Iwate, Ichinoseki, Geibikei	38.9845	141.255667	C17
Reinhardt, 1877	62	Japan, Honshu Is., Fukushima, Urabandai	37.687783	140.1205	C17
	63	Japan, Honshu Is., Kanagawa, Yamakita, Oomatazawa	35.423333	139.005	C18
	64	Taiwan, Taichung County, Heping, Shei-Pa N.P., Huanshan	24.37295	121.310567	C19
*C. occidentale*	65	USA, Washington, Mason County	47.3038	−123.0918	C20
Pilsbry, 1891	66	USA, Washington, Mason County	47.3038	−123.0918	C20
	67	USA, Washington, Mason County	47.3038	−123.0918	C20
	68	USA, Washington, Mason County	47.3038	−123.0918	C20
	69	USA, Washington, Mason County	47.3038	−123.0918	C20
*C.* cf. *pessimum*	70	Russia, Vladivostok, Promorsky Kray	43.193417	132.0511	C21
Pilsbry, 1902	71	Russia, Vladivostok, Promorsky Kray	43.193417	132.0511	C21
	72	Russia, Vladivostok, Promorsky Kray	43.193417	132.0511	C21
	73	Russia, Vladivostok, Promorsky Kray	43.193417	132.0511	C21
	74	Russia, Vladivostok, Promorsky Kray	43.193417	132.0511	C21
	75	Japan, Honshu Is., Iwate, Sarusawa	39.021167	141.292833	C22
*C. stygium*	76	USA, Kentucky, Hart County, Horse Cave (Hidden River Cave)	37.174167	−85.903833	C1
Call, 1897	77	USA, Kentucky, Hart County, Horse Cave (Hidden River Cave)	37.174167	−85.903833	C1
	78	USA, Tennessee, Slit Cave (cave entrance)	36.456833	−85.375067	C1
	79	USA, Tennessee, Slit Cave (cave entrance)	36.456833	−85.375067	C1
*C. tridentatum*	80	Switzerland, St. Gallen, St. Gallen	47.36	9.14	C23
(Risso, 1826)	81	Italy, Upper Adige, near Auer	46.3385	11.3503	C23
	82	France, Britanny, near Lopreden	48.596667	−3.97	C23
*Carychium* sp. 1	83	Bulgaria, Mostovo Village, entrance Gargina Dupka Cave (Rhodopi Mnt.)	41.850667	24.926183	C23
	84	Bulgaria, Mostovo Village, entrance Gargina Dupka Cave (Rhodopi Mnt.)	41.850667	24.926183	C23
	85	Italy, Lombardy, Lago Maggiore, Laveno-Mombello	45.90718	8.6625	C23
	86	Italy, Lombardy, Lago d'Iseo, Vigolo	45.7367	10.0025	C23
	87	Romania, Bacâia	46.0249	23.1734	C23
	88	Georgia, Kakheti Province, Lagodekhi Nature Reserve	41.88	46.31	C23
	89	Georgia, Kakheti Province, Lagodekhi Nature Reserve	41.88	46.31	C23
*Carychium* sp. 2	90	China, Yunnan, Zhongdian County, Shangri-La	27.501017	100.033833	C24
	91	China, Yunnan, Zhongdian County, Shangri-La	27.501017	100.033833	C24
	92	China, Yunnan, Zhongdian County, Shangri-La	27.501017	100.033833	C24
	93	China, Yunnan, Zhongdian County, Shangri-La	27.501017	100.033833	C24
	94	China, Yunnan, Zhongdian County, Shangri-La	27.501017	100.033833	C24
*Carychium* sp. 3	95	China, North Sichuan, Songpan County, Huanglong	32.7373	103.824383	C25
	96	China, North Sichuan, Songpan County, Huanglong	32.7373	103.824383	C25
	97	China, North Sichuan, Songpan County, Huanglong	32.7373	103.824383	C25
***Zospeum*** Bourguignat, 1856			**cave name**		
*Z. alpestre kupitzense*	98	Slovenia, Kamnik-Savinja Alps, Solčava	Ložekarjeva zijalka	Z1	
A. Stummer, 1984	99	Slovenia, Kamnik-Savinja Alps, Solčava	Ložekarjeva zijalka	Z1	
*Z.* cf. *biscaiense* Gómez & Prieto, 1983	100	Spain, Garaimendi, Yurre	Cueva de Otxas*	Z2	
*Z. exiguum*	101	Slovenia, Cerknica, Lož	Križna jama*	Z3	
Kusčer, 1932	102	Slovenia, Cerknica, Lož	Križna jama*	Z3	
	103	Slovenia, Cerknica, Lož	Križna jama*	Z3	
	104	Slovenia, Cerknica, Lož	Križna jama*	Z3	
	105	Slovenia, Cerknica, Lož	Križna jama*	Z3	
*Z. frauenfeldi*	106	Slovenia, Dobrepolje, Podpeč	Podpeška jama*	Z4	
(Freyer, 1855)	107	Slovenia, Dobrepolje, Podpeč	Podpeška jama*	Z4	
	108	Slovenia, Dobrepolje, Podpeč	Podpeška jama*	Z4	
	109	Slovenia, Dobrepolje, Podpeč	Podpeška jama*	Z4	
	110	Slovenia, Dobrepolje, Podpeč	Podpeška jama*	Z4	
*Z. isselianum*	111	Slovenia, Kamnik, Kamniška Bistrica	Jama pod Farjevim plazom	Z5	
Pollonera, 1887	112	Slovenia, Kobarid*, Robič	Turjeva jama	Z6	
	113	Slovenia, Kamnik-Savinja Alps, Šmihel nad Mozirjem	Konečka zijalka	Z7	
	114	Slovenia, Kamnik-Savinja Alps, Šmihel nad Mozirjem	Konečka zijalka	Z7	
	115	Croatia, Kordun, Karlovac, Krnjak, Brebornica Mts.	Jopićeva špilja	Z8	
	116	Croatia, Kordun, Karlovac, Krnjak, Brebornica Mts.	Jopićeva špilja	Z8	
	117	Croatia, Kordun, Karlovac, Krnjak, Brebornica Mts.	Jopićeva špilja	Z8	
	118	Croatia, Kordun, Karlovac, Krnjak, Brebornica Mts.	Jopićeva špilja	Z8	
*Z. obesum*	119	Slovenia, Gradiček, Krška vas	Krška jama*	Z9	
(Frauenfeld, 1854)	120	Slovenia, Gradiček, Krška vas	Krška jama*	Z9	
	121	Slovenia, Gradiček, Krška vas	Krška jama*	Z9	
*Z. pretneri* Bole, 1960	122	Croatia, Gračac, Kesići	Donja Cerovačka špilja*	Z10	
*Z. spelaeum schmidti*	123	Slovenia, Veliki Otok, Postojna	Betalov Spodmol	Z11	
(Frauenfeld, 1854)	124	Slovenia, Loka pri Mengšu	Jama 1 pri Jabljah	Z12	
	125	Slovenia, Loka pri Mengšu	Jama 1 pri Jabljah	Z12	
	126	Slovenia, Loka pri Mengšu	Jama 1 pri Jabljah	Z12	
	127	Slovenia, Loka pri Mengšu	Jama 1 pri Jabljah	Z12	
	128	Slovenia, Loka pri Mengšu	Jama 1 pri Jabljah	Z12	
	129	Slovenia, Krim Region, Gornji Ig	Velika Pasica*	Z11	
	130	Slovenia, Krim Region, Gornji Ig	Velika Pasica*	Z11	
	131	Italy, Trieste, near Gabrovizza San Primo	Grotte d'Ercole	Z11	
	132	Italy, Trieste, near Gabrovizza San Primo	Grotte d'Ercole	Z11	
	133	Italy, Trieste, near Basovizza	Grotte Bac	Z11	
	134	Italy, Trieste, near Basovizza	Grotte Bac	Z11	
	135	Italy, Trieste, near Samatorza	Grotte Azzurra	Z11	
*Z. suarezi*	136	Spain, Cantabria, Novales, La Busta	Cueva del Linar	Z13	
Gittenberger, 1980	137	Spain, Cantabria, Novales, La Busta	Cueva del Linar	Z13	
	138	Spain, Cantabria, Novales, La Busta	Cueva del Linar	Z13	
	139	Spain, Cantabria, Novales, La Busta	Cueva del Linar	Z13	
	140	Spain, Asturias, Inguanzo	Cueva del Bosque/Cueva Inguanzo*	Z14	
	141	Spain, Asturias, Inguanzo	Cueva del Bosque/Cueva Inguanzo*	Z14	
	142	Spain, Bizkaia, San Pedro de Galdames, Barranco de Aranaga	Cueva de los Cuervos	Z15	
	143	Spain, Bizkaia, San Pedro de Galdames, Barranco de Aranaga	Cueva de los Cuervos	Z15	
	144	Spain, Bizkaia, San Pedro de Galdames, Barranco de Aranaga	Cueva de los Cuervos	Z15	
	145	Spain, Castilla y León, Monte Santiago	Cueva de Las Paúles	Z16	
*Z. subobesum*	146	Croatia, Ogulin, Tounj	Tounjčica*	Z8	
Bole, 1974	147	Croatia, Ogulin, Tounj	Tounjčica*	Z8	
	148	Croatia, Ogulin, Tounj	Tounjčica*	Z8	
*Zospeum* sp. 1	149	Spain, Bizkaia, Guipúzcoa Mts., Valle de Araotz	Cueva de Ermita de Sandaili	Z17	
	150	Spain, Bizkaia, Guipúzcoa Mts., Valle de Araotz	Cueva de Ermita de Sandaili	Z17	
	151	Spain, Bizkaia, Guipúzcoa Mts., Valle de Araotz	Cueva de Ermita de Sandaili	Z17	
	152	Spain, Garaimendi, Yurre	Cueva de Otxas	Z17	
	153	Spain, Garaimendi, Yurre	Cueva de Otxas	Z17	
	154	Spain, Garaimendi, Yurre	Cueva de Otxas	Z17	
	155	Spain, Garaimendi, Yurre	Cueva de Otxas	Z17	
	156	Spain, Garaimendi, Yurre	Cueva de Otxas	Z17	
	157	Spain, Bizkaia, Manaria, Urkuleta Valley	Cueva Silibranka II	Z17	
	158	Spain, Bizkaia, Manaria, Urkuleta Valley	Cueva Silibranka II	Z17	
	159	Spain, Bizkaia, Manaria, Urkuleta Valley	Cueva Silibranka II	Z17	
	160	Spain, Bizkaia, Dima, Indusi	Cueva del Cráneo	Z17	
*Zospeum* sp. 2	161	Spain, Castilla y León, Monte Santiago	Cueva de Las Paúles	Z18	
	162	Spain, Castilla y León, Monte Santiago	Cueva de Las Paúles	Z18	
	163	Spain, Castilla y León, Monte Santiago	Cueva de Las Paúles	Z18	
**Outgroup taxa**					
Pythiidae, *Laemodonta cubensis*	164	Bermuda, Hamilton Parish, Walsingham Pond, outside Cliff Cave	32.34773	−64.70965	
Melampodidae, *Microtralia occidentalis*	165	Bermuda, St. George Parish, St. George Island, Lover's Lake	32.36750	−64.70990	
Veronicellidae, *Veronicella cubensis*	166	Bermuda, Devonshire Parish, Winfried Gibbons Nature Reserve, South Road	32.30230	−64.74450	

Data on the morphological identification, taxonomic first description, specimen number (#), sampling locality and evolutionary lineage (EL) are provided. Type locality populations or localities referred to in the phenotype descriptions of *Zospeum* morphospecies are marked by an asterisk.

A conservative delimitation strategy was used to combine the partitions of all five approaches. Since all molecular methods tended to split morphospecies, we decided to risk taxonomic lumping, and classified ELs as the most comprehensive grouping of specimens predicted by any of the five delimitation methods. Thus, the 28 carychiid morphospecies (Figures 1 and 2, Table 1) comprised 43 distinct ELs (Figures 3 and 4, Table 2). In total, 17 morphospecies were each recovered as a single EL (Table 2; ‘matches’). The morphologically distinct, but so far, undescribed *Carychium* sp. 2, *Carychium* sp. 3 (both from China), *Zospeum* sp. 1 and *Zospeum* sp. 2 (both from Cantabrian Mts.) were also genetically revealed as separate clusters. Divergent ELs within a single morphospecies hypothesis were found for eight morphospecies (32%): *Carychium exile mexicanum* (2 lineages), *C. mexicanum* (2), *C. mexicanum costaricanum* (3), *C. noduliferum* (3), *C.* cf. *pessimum* (2), *Zospeum isselianum* (3), *Z. spelaeum schmidti* (2) and *Z. suarezi* (4). Hence, we uncovered at least 20 ELs that could not clearly be distinguished with the initial morphospecies hypotheses (i.e. 47% of all ELs). For *Zospeum isselianum*, *Z. spelaeum schmidti*, *Z. suarezi*, *Z. subobesum* and *Carychium mexicanum costaricanum*, our analysis of topotypic populations enabled us to link a single EL to the initial morphospecies hypothesis (Tables 1 and 2). The three morphospecies, *Zospeum isselianum*, *Z. spelaeum schmidti* and *Z. suarezi* were found over a large geographical area and possessed moderately variable shell phenotypes, two characteristics that make them prime candidates for taxonomic lumping. Specimens of *C. exile mexicanum*, *C. clappi* and *C. stygium* shared closely related barcodes and consequently, were treated as a single taxon (C1). The European *Carychium* sp. 1 (C23) needs further consideration since results of the barcoding approach and phylogenetic reconstruction were not congruent. Thus, two ELs (C1 and C23) included more than one morphospecies each.

**Table 2:** Integrative identification of evolutionary lineages in Carychiidae

**EL**	**cross-validation with MA**
C1	includes *C. clappi*, some specimens of *C. exile mexicanum* and *C. stygium*
C2	matches *C. exiguum*
C3	matches *C. exile*
C4	lumped with specimens from C1 as *C. exile mexicanum*
C5	matches *C. floridanum*
C6	matches *C.* cf. *schlickumi*
C7	matches *C. ibazoricum*
C8	matches *C. lederi*
C9	lumped with C10 as *C. mexicanum*
C10	lumped with C9 as *C. mexicanum*
C11	regarded as *C. costaricanum*; lumped with C12 + C13 as *C. mexicanum costaricanum*
C12	lumped with C11 + C13 as *C. mexicanum costaricanum*
C13	lumped with C11 + C12 as *C. mexicanum costaricanum*
C14	matches *C. minimum*
C15	matches *C. nannodes*
C16	matches *C. nipponense*
C17	lumped with C18 + C19 as *C. noduliferum*
C18	lumped with C17 + C19 as *C. noduliferum*
C19	lumped with C17 + C18 as *C. noduliferum*
C20	matches *C. occidentale*
C21	lumped with C22 as *C.* cf. *pessimum*
C22	lumped with C21 as *C.* cf. *pessimum*
C23	includes *C. tridentatum* and *Carychium* sp. 1
C24	matches *Carychium* sp. 2
C25	matches *Carychium* sp. 3
Z1	matches *Z. alpestre kupitzense*
Z2	matches *Z.* cf. *biscaiense*
Z3	matches *Z. exiguum*
Z4	matches *Z. frauenfeldi*
Z5	lumped with Z6 + Z7 as *Z. isselianum*
Z6	regarded as *Z. isselianum*; lumped with Z5 + Z7 as *Z. isselianum*
Z7	lumped with Z5 + Z6 as *Z. isselianum*
Z8	matches *Z. subobesum*; specimens 146–148 identified as *Z. isselianum*
Z9	matches *Z. obesum*
Z10	matches *Z. pretneri*
Z11	regarded as *Z. spelaeum schmidti*; lumped with Z12 as *Z. spelaeum schmidti*
Z12	lumped with Z11 as *Z. spelaeum schmidti*
Z13	lumped with Z14, Z15 + Z16 as *Z. suarezi*
Z14	regarded as *Z. suarezi*; lumped with Z13, Z15 + Z16 as *Z. suarezi*
Z15	lumped with Z13, Z14 + Z17 as *Z. suarezi*
Z16	lumped with Z13, Z14 + Z15 as *Z. suarezi*
Z17	matches *Zospeum* sp. 1
Z18	matches *Zospeum* sp. 2

Overview of cross-validated evolutionary lineages (EL) and initial morphospecies assignments (MA). ‘matches’ = direct match between EL and initial MA; ‘lumped’ = more than one EL in initial MA; ‘includes’ = EL includes more than one morphologically delineated taxon; ‘regarded as’ = used for a ‘lumped’ EL in case type locality populations have been analyzed.

### Phylogenetic tree hypothesis

**Figure 5:**
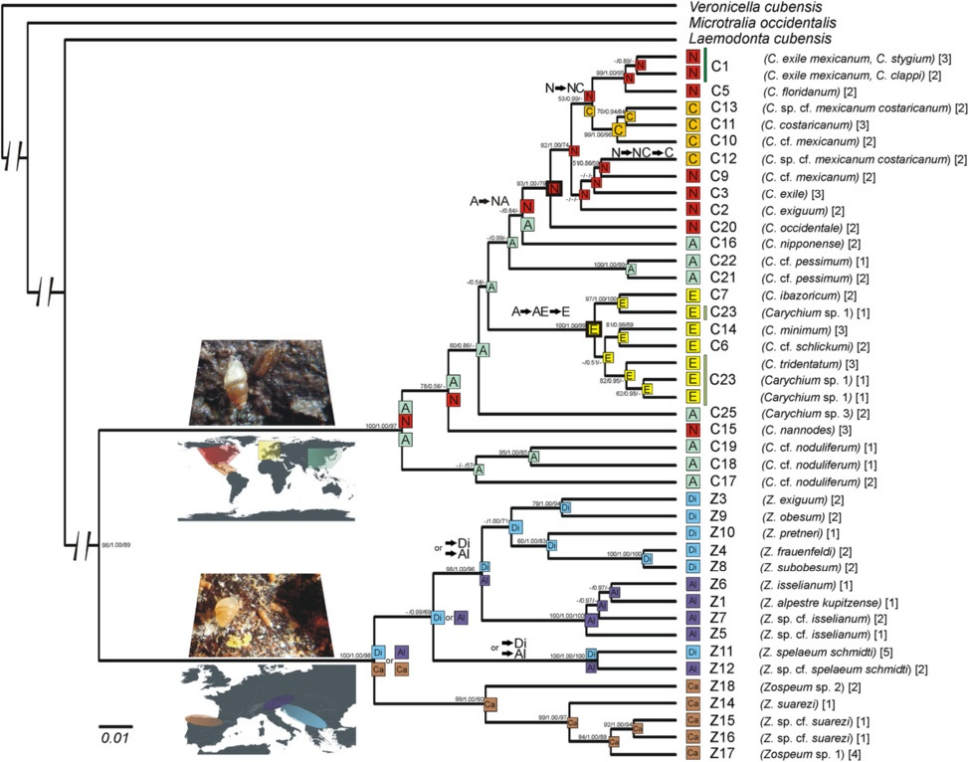
**Phylogenetic hypothesis of Carychiidae.** A consensus tree representing phylogenetic relationships of 22 *Carychium* and 16 *Zospeum* evolutionary lineages (EL) is illustrated. Statistical support is provided at the branches in the following order: Maximum Composite Likelihood bootstrap / Bayesian posterior probability / Maximum Likelihood bootstrap. For each EL, integrative decisions on the lineage assignments (see result section) and the number of individuals analyzed is given in brackets. Codes for the distribution and geographical range evolution are: North America (N; red), Central America (C; orange), Asia (A; cyan), Europe (E; yellow), Dinaric Alps (Di; blue), Alps (Al; purple) and Cantabrian Mountains (Ca; brown). Equally possible range scenarios are separated by an ‘or’. Arrows illustrate the directionality of range shifts. Geographic information and living specimens are shown at the branches giving rise to *Carychium* and *Zospeum*, respectively.

Phylogenetic relationships of 38 ELs based on 1210 bp of nuclear (H3) and mitochondrial sequence data (COI and 16S) were estimated with three different phylogenetic inference methods. Maximum Likelihood (ML), Maximum Composite Likelihood (MCL) and Bayesian inference (BI) yielded mainly congruent results (Figure 5). Lineage assignments correspond to the identification scheme for ELs described in the previous chapter however, with the following exceptions: morphologically unrecognized ELs, for which topotypic populations of the same morphospecies were analysed, were marked with ‘sp. cf.’ and the morphospecies name (e.g. Z16, *Z.* sp. cf. *suarezi*). The EL of the same morphospecies, which included specimens from the type locality or from localities used for the phenotype description, was named after the morphospecies hypothesis (e.g. Z14, *Z. suarezi* from Cueva Inguanzo referred to in the first description [31]). Due to the molecular distinctiveness of *C. costaricanum* (C11) from its type locality [32], we address this taxon at the species level.

The morphologically and ecologically defined designation into *Carychium* and *Zospeum* was supported by molecular data (MCL: 100; PP: 1.00; ML ≥ 97). All analysed mitochondrial ELs (C1-25 and Z1-18, Figure 5) were monophyletic after incorporation of ncDNA with the exception of *Carychium* sp. 1 (C23). However, sister relationship between lineages were not resolved entirely. Only the relationship of one individual of *Carychium* sp. 1 to *C. ibazoricum* (C7) was highly supported (97; 1.00; 100). Another cluster formed by two lineages of *Carychium* sp. 1 and *C. tridentatum* was only weakly supported (82; 0.95; -).

Except for *C. nannodes*, the North + Central (N+C) American taxa can be traced back to one most recent common ancestral lineage (93; 1.00; 79). The only *Carychium* taxon with a distribution along the West Coast of North America, *C*. *occidentale* forms the sister group to all other taxa in North and Central America (92; 1.00; 79). Extant European *Carychium* have descended from a single lineage (100; 1.00; 99).

Well resolved divergence events for ELs of *Carychium* comprised: i) a clustering of C1 + C5, including the morphospecies *C. exile mexicanum*, *C. stygium*, *C. clappi* and *C. floridanum* (99; 1.00; 95), ii) a clade formed by three C-American lineages C10 + C11 + C13 and the morphospecies *C.* cf. *mexicanum*, *C. costaricanum* and *C.* cf. *mexicanum costaricanum* (99; 1.00; 96) and iii) the sister group relationship of *C. minimum* (C14) and *C.* cf. *schlickumi* (C6) (81; 0.96; 89).

Support values for diversification events within *Zospeum* were high. *Zospeum* from the Cantabrian Mountains were monophyletic (99; 1.00; 90). A sister group relationship between *Zospeum* sp. 2 (Z18) and a clade comprising the morphospecies *Z. suarezi* and *Zospeum* sp. 1 (Z17) was revealed (99; 1.00; 97). Within this clade, three distinct ELs form the morphospecies *Z. suarezi* (Z14-Z16), which was paraphyletic with respect to *Zospeum* sp. 1 (94; 1.00; 89). The widely distributed morphospecies *Z. spelaeum schmidti* was monophyletic but contained two deeply separated ELs (100; 1.00; 100). Monophyly between the Alpine *Z. isselianum* (Z5-Z7) and *Z. alpestre kupitzense* (Z1) was strongly supported (100; 1.00; 100). The relationship between the Dinaric morphospecies *Z. exiguum*, *Z. obesum*, *Z. pretneri*, *Z. frauenfeldi* and *Z. subobesum* received only partial support (−; 1.00; 71). Sister group relationships between the Dinaric *Z. frauenfeldi* (Z4) and *Z. subobesum* (Z8) (100; 1.00; 100) and between *Z. exiguum* (Z3) and *Z. obesum* (Z9) (79; 1.00; 94) were well resolved. Remarkably, the Dinaric *Z. isselianum* (specimens 115–118, Z8, see Figure 4) did not cluster with other Alpine *Z. isselianum* lineages (Z5-Z7) but fell within the Dinaric clade. Dinaric *Z. isselianum* were probably misidentified. They clustered with *Z. subobesum* (Z8) for which topotypic specimens have been analyzed (Figures 4 and 5, Tables 1 and 2).

### Biogeographical reconstruction

Geographical range evolution of Carychiidae was reconstructed to evaluate geographical transitions and the presence or absence of geographically monophyletic clades. Results are shown only for the model where taxa inhabit a maximum of two regions (continents or mountain ranges). Differences to alternative models, where the maximum range was allowed to encompass all ranges (3 or 4), were largely restricted to basal nodes. However, in some cases these scenarios resulted in extremely wide ancestral ranges which seems biologically unreasonable. Generally, geographical proximally distributed taxa were closely related. Only few colonization events were discovered. The range reconstruction of the most likely (conservative) scenario for the root node of *Carychium* revealed an ‘Asian’ (A) or ‘Asian + North American’ (A+N) origin (Figure 5).

The oldest diversification events within *Carychium* are characterized by relatively short branches with low statistical support, giving rise mostly to deep Asian lineages. Central America could have been colonized by more than one lineage. The European branch originated out of a long separately evolving lineage. We further tested two alternative tree hypotheses for *Carychium* in a model selection framework: i) constrained monophyletic Asian *Carychium* and ii) constrained monophyletic American *Carychium* (see Additional file 1). Both constraints received higher support than the unconstrained phylogenetic hypothesis. Monophyletic Asian and/or American lineages should not be ruled out (Table 3).

**Table 3:** Model selection results of alternative phylogenetic hypothesis testing

**hypothesis**	**ΔAICM**	**ln BF**_**PS**_	**ln BF**_**SS**_
monophyletic American *Carychium*	0.00	0.00	0.00
monophyletic Asian *Carychium*	−14.07	17.24	17.66
unconstrained phylogeny	−18.96	44.86	45.14
monophyletic Alpine *Zospeum*	−157.41	85.25	85.81
monophyletic Dinaric *Zospeum*	−345.81	191.58	190.53

Results of the model selection approach to test the monophyly of geographically closely distributed taxa. Hypotheses are ranked according to model fit. AICM = modified Akaike Information Criterion; BF_PS_ = Bayes Factor based on path simulation sampling; BF_SS_ = Bayes Factor based on stepping stone sampling. A ΔAICM >7 and a ln BF > 2.3 are seen as strong model support.

The geographic origin of *Zospeum* could not be unambiguously resolved (Figure 5). Our reconstructions equally supported an ancestral distribution in the ‘Cantabrian Mountains + Alps’ or ‘Cantabrian Mountains + Dinaric Alps’. In comparison to *Carychium*, *Zospeum* arrived much earlier in Europe. Only two colonization events were discovered: Both lineages of *Z. spelaeum schmidti* were geographically restricted to the Alps (Z12) and the Dinaric Alps (Z11), respectively. Moreover, the sister clade (comprised of all other Dinaric and Alpine taxa) demonstrated a similar pattern with separate colonization of the Alps or Dinaric Alps; depending upon the ancestral state. We tested our phylogenetic hypothesis against two constrained hypotheses for *Zospeum*: i) monophyletic Dinaric *Zospeum* and ii) monophyletic Alpine *Zospeum*. Both scenarios received considerably lower support than the unconstrained hypothesis (Table 3).

### Temporal dynamics of lineage diversification

**Figure 6:**
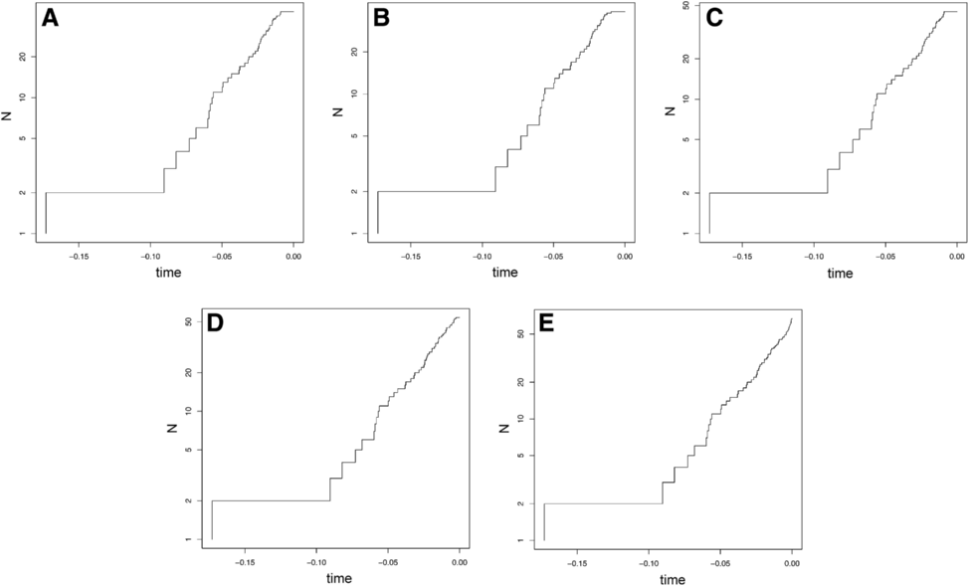
**Lineage through time plots (LTT-plot).** The diversification of lineages (y-axis; cumulative number of lineages) through time (x-axis; relative time estimates) is plotted in five different ways according to the delimitation of evolutionary lineages: **A:** 3.2% threshold value partition. **B:** Automatic Barcoding Gap Detection (ABGD) partition. **C:** Statistical Parsimony (SP) partition. **D:** General Mixed Yule-Coalescent single (GMYCs) partition. **E:** General Mixed Yule-Coalescent multiple (GMYCm) partition.

By fitting different models of diversification using a maximum likelihood approach, temporal dynamics of the diversification of lineages, such as rate shifts due to historic climatic or tectonic changes, were investigated. To account for uncertainties in species delimitation, the analyses were performed on each of the five trees produced by the genetic delimitation approaches (threshold, ABGD, SP, GMYCs and GMYCm). A constant rate model of diversification was preferred for the GMYCm tree (Figure 6, Table 4), whereas a rate-variable model (yule2rate) provided a better fit for all other trees (but not significantly in case of the GMYCs tree). A two-rate pure-birth model best explained the data for the threshold, ABGD and SP trees (Table 4) with a recent decrease (relative shift time approx. -0.01) of the speciation rate.

**Table 4:** **Model results for the temporal lineage diversification of *Carychium *and *Zospeum***

**model**	**P**	**df**	**mtype**	**LH**	**r1**	**r2**	**a**	**xp**	**k**	**st**	**AIC**	**ΔAIC**
**threshold**												
yule2rate	r1, r2, st	3	RV	212.45	30.83	6.04	n.a.	n.a.	n.a.	0.0117	−418.90	0
DDL	r1, k	2	RV	208.09	37.57	n.a.	n.a.	n.a.	68.32	n.a.	−412.18	6.73
pureBirth	r1	1	RC	206.58	23.71	n.a.	n.a.	n.a.	n.a.	n.a.	−411.16	7.74
DDX	r1, xp	2	RV	206.61	26.44	n.a.	n.a.	0.04	n.a.	n.a.	−409.21	9.69
bd	r1, a	2	RC	206.58	23.71	n.a.	0.00	n.a.	n.a.	n.a.	−409.16	9.74
**ABGD**												
yule2rate	r1, r2, st	3	RV	188.50	30.78	3.65	n.a.	n.a.	n.a.	0.0142	−371.00	0
DDL	r1, k	2	RV	182.55	39.38	n.a.	n.a.	n.a.	53.63	n.a.	−361.10	9.90
pureBirth	r1	1	RC	180.28	21.97	n.a.	n.a.	n.a.	n.a.	n.a.	−358.56	12.44
DDX	r1, xp	2	RV	180.40	27.75	n.a.	n.a.	0.08	n.a.	n.a.	−356.81	14.20
bd	r1, a	2	RC	180.28	21.97	n.a.	0.00	n.a.	n.a.	n.a.	−356.56	14.44
**SP**												
yule2rate	r1, r2, st	3	RV	226.28	31.07	4.65	n.a.	n.a.	n.a.	0.0096	−446.56	0
DDL	r1, k	2	RV	221.20	36.80	n.a.	n.a.	n.a.	78.07	n.a.	−438.40	8.16
pureBirth	r1	1	RC	220.00	24.58	n.a.	n.a.	n.a.	n.a.	n.a.	−438.01	8.56
bd	r1, a	2	RC	220.00	24.58	n.a.	0.00	n.a.	n.a.	n.a.	−436.01	10.56
DDX	r1, xp	2	RV	220.00	24.58	n.a.	n.a.	0.00	n.a.	n.a.	−436.01	10.56
**GMYCs**												
yule2rate	r1, r2, st	3	RV	285.59	31.68	9.37	n.a.	n.a.	n.a.	0.0040	−565.17	0
pureBirth	r1	1	RC	283.47	29.02	n.a.	n.a.	n.a.	n.a.	n.a.	−564.93	0.24
DDX	r1, xp	2	RV	283.78	20.50	n.a.	n.a.	−0.11	n.a.	n.a.	−563.56	1.61
bd	r1, a	2	RC	283.52	26.48	n.a.	0.15	n.a.	n.a.	n.a.	−563.04	2.14
DDL	r1, k	2	RV	283.47	29.44	n.a.	n.a.	n.a.	1927.73	n.a.	−562.94	2.23
**GMYCm**												
bd	r1, a	2	RC	385.03	16.10	n.a.	0.74	n.a.	n.a.	n.a.	−766.06	0
DDX	r1, xp	2	RV	384.37	12.05	n.a.	n.a.	−0.35	n.a.	n.a.	−764.75	1.31
yule2rate	r1, r2, st	3	RV	385.23	30.56	71.35	n.a.	n.a.	n.a.	0.0042	−764.47	1.60
pureBirth	r1	1	RC	381.35	35.94	n.a.	n.a.	n.a.	n.a.	n.a.	−760.69	5.37
DDL	r1, k	2	RV	381.35	35.94	n.a.	n.a.	n.a.	11.20x10^-3^	n.a.	−758.69	7.37

Results are shown for the five different molecular delimitation approaches. Model = model name; P = parameters included, df = degrees of freedom, mytpe = model type; rate-constant (RC) or rate-variable (RV), LH = model log likelihood, r1 = initial diversification rate, r2 = second diversification rate, a = extinction fraction, xp = the x-parameter from the DDX model, k = the k-parameter from the DDL model, st = shift-time, AIC = Akaike Information Criterion, ΔAIC = delta AIC, the difference in AIC scores between given model and the overall best-fit model, n.a. = not available.

### Bioclimatic niche differentiation

Bioclimatic niche modeling was performed to study niche differences between two well supported *Carychium* clades and to identify potential climatic factors responsible for lineage persistence and diversification. Maxent models based on occurrence data of European and North + Central American *Carychium* behaved realistically and were statistically well supported (AUC_Europe_: 0.769 ± 0.033; AUC_NorthCentralAmerica_: 0.881± 0.016) (see Additional file 2). Bioclimatic niche models for native *Carychium* taxa in America and in Europe, respectively, generally predicted wide areas of suitable habitat (see Additional file 2A, B). The American model was characterized by a sharp longitudinal transition from suitable habitats in the East to unsuitable conditions in the West. Visual inspection of the geographical distribution of all 19 bioclimatic variables throughout this region identified diurnal range (bio2) to best explain the observed pattern (see Additional file 3). Modeling results for Europe highlighted a continuous region of high habitat suitability, stretching from the Mediterranean Sea to Northern Scandinavia and from the Atlantic Coast to the Black Sea.

Compared to the niche model of the native clade in Europe, the projected model for America suspected European *Carychium* to inhabit regions at lower and higher latitude (see Additional file 2C). Variable degrees of predicted habitat suitability were modeled for populations of non-native European *Carychium minimum* (CM) and *C. tridentatum* (CT) populations in North America (see Additional files 2C and Additional file 4). The modeled latitudinal distribution in Eastern North America was interrupted by a large territory of unsuitable bioclimatic conditions. Similar to the native American clade, this phenomenon could be attributed to changes in the variable states of diurnal range (see Additional file 3). Projection of the North and Central American bioclimatic envelope onto Europe largely corresponded with the distribution range of native *Carychium* taxa (see Additional file 2D). However, a slight shift towards more Mediterranean (to the South) and continental conditions (to the East) was visible.

Bioclimatic niche envelopes of both *Carychium* clades indicated moderate niche overlap in America (D: 0.3878, I: 0.6885, RR: 0.5998; Table 5). Niche overlap was considerably higher on the European continent (D: 0.6162, I: 0.8570, RR: 0.6589). Niche breadth, i.e. the flatness of the distribution of suitability scores (D. Warren, pers. comm.) differed between both clades with the American clade demonstrating lower values of niche breadth throughout both regions (B1_America_ < B1_EuropeOntoAmerica_; B1_AmericaOntoEurope_ < B1_Europe_) (Table 6).

**Table 5:** **Niche overlap within *Carychium *in North America and Europe**

	**Niche overlap statistic**		
	D	I	relative rank
Overlap in North America	0.3878	0.6885	0.5998
Overlap in Europe	0.6162	0.8570	0.6589

Habitat suitability maps were calculated based on species occurrences in each region (Europe and North America) independently and projected into the other region (North America and Europe, respectively).

**Table 6:** Niche breadth statistic for native and projected models

	**Niche breadth**
America	0.326
Europe onto America	0.374
Europe	0.549
America onto Europe	0.472

Niche breadth for *Carychium* species in Europe and North America as well as according to the projected niches into the other region is shown.

## Discussion

### Unrecognized evolutionary lineages

While most species concepts view a species as ‘a separately evolving metapopulation lineage’ [33], they disagree on which characters should be applied to organize this speciation continuum [33]. In the case of the microgastropod taxon Carychiidae, variable environmental conditions can lead to phenotypic variability (e.g. lineages inhabit large geographical regions), whereas relatively stable environments can lead to morphological stasis (e.g. cave endemic lineages) [34-38]. Given that traditional taxonomic classifications completely relied upon characters of the mature shell, unrecognized ELs had to be expected.

Integration of molecular data in a conservative delimitation approach revealed that the 28 analyzed morphospecies actually comprise 43 distinct ELs. From our results, three different taxonomic scenarios can be distinguished: i) ELs directly matching the morphospecies, including morphologically delimited, but so far, undescribed taxa (21 lineages), ii) distinct ELs being lumped into a single morphospecies (8 morphospecies encompassing 20 lineages), and iii) single ELs including more than one morphospecies hypothesis (4 morphospecies, 1 undescribed taxon, 2 lineages). This suggests that basing carychiid taxonomic delineations only on conchological characters may be inadequate. We emphasize that vague taxonomic (under-) descriptions and semantic tradition, with morphospecies concepts often only referring to a few specimens, have widely neglected aspects such as intraspecific shell variability. In congruence with overlapping, interspecific shell dimensions, this has led to a vague situation in carychiid taxonomy and systematics.

Carychiidae appear to still harbor a considerable amount of undiscovered diversity, especially in biogeographic regions that are underexplored due to political strife, non-access and challenging geography, such as Asia or Central America. For example, East Asian *C. noduliferum, C.* cf. *pessimum* and the Central American *C. mexicanum* and *C. mexicanum costaricanum* morphospecies for which, at least one unrecognized EL was discovered*.* In *Zospeum*, similar as in other cave animals [39-41], ELs are likely to be morphologically cryptic, possibly due to morphological stasis.

On the other hand *Carychium exile mexicanum* most likely served as a ‘taxonomic lumping bin’ built up by the frequent nomenclatural intermixture of ELs of the morphospecies *C. stygium*, *C. clappi*, *C. floridanum*, *C. mexicanum* and *C. mexicanum costaricanum* (e.g. [42-46]). The relevant taxonomic literature is puzzling and contains contradictory statements [42]. In general, individual morphospecies within the *C. exile mexicanum + C. stygium* + *C. clappi* EL showed highly similar barcode sequences and are thus, difficult to distinguish by molecular delimitation methods, suggesting that these are probably in the process of speciation [18]. A similar complex situation is evident for *C. tridentatum* and *Carychium* sp. 1, which still need further taxonomic investigation. An alternative explanation for the clustering of morphologically distinguishable taxa may be an artifact created by our conservative genetic delimitation strategy. However, if we expect these ‘true’ species to be artificially lumped into a single EL, we may equally expect other ELs to include even more ‘true’ species, e.g. as indicated by the two GMYC models.

Based on our taxonomic investigations, we suggest that apparently widespread and assumedly variable *Zospeum* morphospecies must be revised. Moreover, since several *Zospeum* spp. are already listed as vulnerable or endangered [47], we anticipate high conservation value amidst cave-endemics. Furthermore, we recommend that future molecular analyses should focus on populations collected at the type localities, to link an EL with the historic morphospecies hypothesis. A more comprehensive geographic sampling would most likely uncover yet even more unrecognized ELs.

### Diversification of evolutionary lineages

Appropriate taxon sampling is crucial for the reconstruction of phylogenetic relationships [30]. Our taxon sampling not only covered large parts of the known Holarctic distribution of the Carychiidae but we integrated molecular data to uncover morphologically unrecognized ELs. As expected, the geographic evolution of Carychiidae suggests that the majority of ELs in a geographic area are phylogenetically more closely related than taxa between distant regions (i.e. continents / mountain ranges). This general pattern is most obvious for the European *Carychium* and Cantabrian *Zospeum*. After the initial colonization of new areas due to rare (long-distance) passive dispersal, the ancestral lineages diversified *in situ.*

Based on five different genetic delimitation approaches, our analyses of diversification modes provided mixed results, and in some cases, indicate that diversification rates may have changed over time. While the two GMYC models favored a constant-rate diversification model, the three more conservative genetic delimitation strategies (threshold, ABGD and SP) point to a relatively recent rate slowdown. Such a rate shift can be interpreted in different ways. First, a decrease in the rate of speciation may be due to diversity dependence, e.g. a niche-filling process, where new species reduce the probability of future speciation events [48]. Second, incomplete taxon sampling can result in a spurious rate slowdown due to an overrepresentation of deeper nodes [49]. Third, since speciation is a continuous process, relatively young divergence events are likely to remain unobserved. This effect has recently been described as ‘protracted speciation’ [50]. Given that the more conservative delimitation approaches are likely to underestimate the number of ELs, we suggest that the observed pattern is more likely an artifact than an actual slowdown. The other two causes may be difficult to distinguish as they can lead to similar patterns, but we suggest protracted speciation as the most likely cause here. First, while taxon sampling in *Carychium* and *Zospeum* is indeed incomplete, several of the missing morphospecies in fact seem to belong to ELs included in this study (for example, *C. mariae* and *C. riparium*; [19] and Jochum & Weigand, unpublished data). Second, the very recent shift in diversification rates is expected under protracted speciation, but could result from incomplete taxon sampling only if the missing species were those that have originated most recently. After omitting the youngest 5th percentile of total branch lengths (i.e. excluding the most recent 5% of the evolutionary history), a constant-rate model is also favored for the threshold, ABGD and SP partitions (data not shown). This not only highlights the very recent timing of the rate shift (consistent with protracted speciation), but it also shows that no additional rate shifts throughout the evolutionary history of Carychiidae have occurred, suggesting that major environmental changes did not affect the rate of diversification of this clade.

The geographic evolution of *Carychium* implies an Asian or Asian + North American origin. Asian *Carychium* are the genetically most distinct. Since high regional genetic diversity of a taxon can be taken as evidence for lineage persistence in ancestral areas [51], this provides support for an out-of-Asia hypothesis. Niche models suggest significant overlap and occupancy of a wide range of environmental conditions among two geographically and phylogenetically distant *Carychium* clades (Europe and America). There is, however, no evidence for an intermixture of the East and West North American *Carychium* lineages. Thus, larger-scale bioclimatic factors are likely to have been of lesser importance than microhabitat conditions in the diversification of *Carychium*. Nonetheless, differences in the larger-scale bioclimatic niche can result from independent evolutionary histories in isolated biomes and, affect the marginal distribution of lineages. For example diurnal range patterns, an important factor for North American *Carychium*, might affect the colonization of habitats featuring a high variation between day and night. Temperature extremes, moisture and relative humidity levels in typical microhabitats occupied by *Carychium* (e.g. moist leaf-litter, crevices or superficial subterranean habitats) are much less pronounced than those on the surface [52-54]. In cases where the temperature diurnal range is high, microhabitats will not be able to stabilize a given condition.

Since the available bioclimatic layers do not cover subterranean habitats, the influence of bioclimatic parameters on the distribution and diversification of *Zospeum* could not be addressed. The ancestral area reconstruction indicated the ‘Cantabrian Mountains + Alps’ or ‘Cantabrian Mountains + Dinaric Alps’ as the ancestral area. We identified two independent colonizations of the Alps and/or the Dinaric Alps but the geographical directionality of these colonizations could not yet be deduced. The incorporation of a recently (re)discovered Asian cf. *Zospeum* from Chinese and South Korean caves (RS and [55]) may provide further insight into the evolutionary history of *Zospeum*.

The diversification of *Zospeum* is characterized by rare, long-distance colonization events with *in situ* (mountain range) radiations into several (sometimes morphologically cryptic) lineages, occupying isolated cave systems. Trogloxene cave animals like bats and cave crickets, returning to the surface periodically, represent potential vectors for the passive dispersal of cave-dwelling microgastropods [56,57]. Such an allopatric diversification without phenotypic (and perhaps ecological) divergence is referred to as morphostatic radiation [58] or non-adaptive radiation [59]. Once established at a new locality, ancestral populations may reach adjacent habitats by floating via underground drainage systems or by active subsurface migration [40]. As an example, seasonal flooding events are known triggers for the wash-out of the cave salamander *Proteus anguinus*[60]. As has been shown for human-dispersed transatlantic populations of *Carychium*[26,27], the transportation of only a few hermaphroditic individuals is needed for a successful population foundation. Finally, the remarkably wide distribution of the Cantabrian *Zospeum* sp. 1 with the presence of identical DNA barcodes in four distant caves (up to 30 km apart) suggests recent long-distance dispersal and merits further investigation.

In contrast to the aforementioned, dispersal colonization, cave lineages can arise from multiple diversification events of surface populations via vicariance colonization [56,61]. The geographic distribution of extant *Zospeum* allows room for speculation about the maximum age and evolutionary history for this group. All caves inhabited by European *Zospeum* are embedded in sediments of the Alpine belt initially formed during the Early Cenozoic Alpine orogeny [62,63]. Our most parsimonious assumption suggests that European *Zospeum* originated no earlier than the beginning of the Early Cenozoic (approximately 65 mya), coinciding with the beginning of the Alpine orogeny. Their last common ancestor (LCA) could have descended from the non-cave-dwelling *Carychiopsis*, for which fossils are known since the European Paleocene (66–56 mya) [64-66].

## Conclusions

Carychiidae harbor a substantial number of morphologically unrecognized ELs. In particular, several of the assumedly widespread cave-dwelling *Zospeum* as well as Asian and Central American *Carychium* species resulted from past taxonomic lumping. Future studies should focus on specimens from type localities to link the ELs with the initial phenotype descriptions. Rare long-distance colonization and *in situ* radiations within the newly inhabited areas, i.e. continents (*Carychium* spp.) and mountain ranges (*Zospeum* spp.) represent likely diversification processes in the Carychiidae. However, East Asia provides a notable exception, exhibiting high regional genetic diversity formed by several distinct *Carychium* lineages: indicating a potential origin for *Carychium* and a potentiality for all the Carychiidae.

Although global climatic conditions could influence distribution, microhabitat structure most likely determined local presence and promoted allopatric diversification. Land invasion and desiccation avoidance by the LCA of Carychiidae could have been achieved by colonizing aphotic, permanently humid microenvironments. The occurrence of a true, cave-dwelling lineage (*Zospeum)* and sporadically-observed cave populations in *Carychium* only demonstrate ecological extremes for survival during the adaptive shift onto land. Shallow subterranean habitats, providing a connection between surface and subsurface realms, could well have promoted ecological transitions within the Carychiidae. Future species-specific ecological studies will allow the identification of micro-environmental parameters shaping the distribution and promoting lineage diversification.

## Methods

### Sampling and identification

In total, 166 individuals were collected during the years 2007–2012 (Table 1). Specimens were immediately stored in 70–99 % ethanol after collection. Our dataset comprises 28 morphologically-described (sub-) species (referred to as morphospecies) of the Carychiidae, including 18 *Carychium* (Figure 1) and 10 *Zospeum* taxa (Figure 2). Additional data were retrieved from a previous DNA barcoding study [18]. Initial taxonomic assignments are based upon conchological characters using taxonomic first descriptions, expert opinions (AJ and AMW *Carychium*+*Zospeum*; E. Gittenberger and R. Bank [European *Carychium*; Y. Kano [Japanese *Carychium*; RS and C.E. Prieto *Zospeum*) and relevant taxonomic keys (e.g. [42,46,67-69]). Morphospecies assignments are marked with ‘cf.’ in case of juvenile specimens or tenuous morphological characteristics. In particular, the specimens 27–29 from Epirus (Greece) do not match any description of extant taxa but very much resemble *C. schlickumi* described from the Pliocene [70].

### DNA extraction, PCR and sequencing

DNA extraction was carried out on ethanol-preserved individuals using the DNeasy Blood and Tissue Kit (Qiagen, Hilden, Germany) protocol. Shell and visceral material were removed to lower cross-contamination risk. Polymerase chain reactions (PCR) were performed to amplify nuclear Histone 3 (H3), a partial fragment of the mitochondrial 16S rRNA (16S) and the DNA barcoding fragment of the mitochondrial-encoded Cytochrome C Oxidase Subunit I (COI). For COI, we used the standard invertebrate primer pair LCO1490 – 5’-GGT CAA CAA ATC ATA AAG ATA TTG G-3’ and HCO2198 – 5’-TAA ACT TCA GGG TGA CCA AAA AAT CA-3’ [71]. Each 25 μL PCR mixture included 1 μL (10 pmol) of each primer, 2.5 μL 10x PCR buffer, 2 μL (100 mM) MgCl_2_, 0.3 μL (20 mM) dNTPs, 0.3 μL *Taq*-polymerase, 0.25 μL (0.5 M) tetramethylammonium chloride, 1.5 μL (10 mg / mL) bovine serum albumin, 11.15 μL ddH_2_O and 5 μL template DNA. PCR cycles were run at the following conditions: 1 min at 95°C, followed by 30 cycles of 30 s at 95°C, 30 s at 52°C and 30 s at 72°C, and finally, 3 min at 72°C. For 16S, we used the same PCR conditions and the primer pair 16S-H – 5’-CGC CTG TTT ATC AAA AAC AT-3’ and 16S-R – 5’-CCG GTC TGA ACT CAG ATC ACG T-3’ [72]. For H3, we used the degenerated primer pair H3-F – 5’-ATG GCT CGT ACC AAG CAG AC(ACG) GC-3’ and H3-R – 5’-ATA TCC TT(AG) GGC AT(AG) AT(AG) GTG-3’ [73]. In principle, the same PCR conditions have been used for the amplification of the H3 marker. Modifications contain: the use of 0.1 μL (20 mM) dNTPs, 0.14 μL *Taq*-polymerase and no tetramethylammonium chloride and bovine serum albumin. PCR cycles were run at the following conditions: 5 min at 95°C, followed by 34 cycles of 30 s at 95°C, 25 s at 52°C and 45 s at 72°C, and finally, 5 min at 72°C. Visualization of single PCR products was performed on a 1.4% agarose gel. They were cleaned using the GeneJET PCR Purification Kit (Fermentas, St. Leon-Rot, Germany). In cases multiple PCR products were detected, the QIAquick Gel Extraction protocol (Qiagen) was used. PCR products were bidirectionally sequenced using the PCR primer pair (5 pmol) and the BigDye® Terminator v.3.1 Cycle Sequencing Kit (Applied Biosystems, Inc.) on an ABI 3730 xl capillary sequencer following the manufacturer’s instructions.

### Molecular delimitation strategies

COI sequences of all 166 individuals were aligned using MAFFT 6.814 [74] implemented in the Geneious software under the G-INS -i algorithm proposed for less than 200 sequences with global homology. Ambiguous characters (Ns) were treated as missing data. The alignment was further modified by manual primer deletion and a 3’ and 5’ trimming conducted with GBLOCKS [75]. The length of the final alignment was 607 bp. DNA barcodes are deposited in the BOLD project ‘Barcoding Carychiidae microsnails [BARCA]’.

Initial morphospecies assignments were tested using five genetic delimitation approaches: DNA barcoding via a threshold value [76], the Automatic Barcoding Gap Detection (ABGD) method [77], the General Mixed Yule-Coalescent single (GMYCs) and multiple (GMYCm) models [78,79] and Statistical parsimony network analysis (SP) [80]. A threshold value of 3.2% K2P genetic distance was used to separate intra- and interspecific variability in Carychiidae, as recently established by Weigand et al. [18]. The ABGD method separates DNA sequences based on an automatic procedure of barcode gap discovery. Three user defined input variables are requested: The minimum (Pmin) and maximum intraspecific variability (Pmax), which refer to the area were the barcode gap should be detected; and the minimum gap width (X) which relates to the sensitivity of the method to gap width. We tested model combinations of X ranging from 0.01 to 0.9 with Pmax of 0.001 and 0.9, respectively. All runs were performed using Kimura (K89) genetic distances and 50 screening steps. The GMYC delimitation method combines phylogenetic and phylogeographical approaches to estimate the number of well-separated entities in a sample. It uses an ultrametric input tree to define partitions according to transitional points between speciation and coalescence within species rates. A model based on a single (GMYCs) or multiple (GMYCm) threshold values can be tested. Analyses and model comparisons were performed in the R package ‘Splits’ using the ‘gmyc’ and ‘compare’ functions. The ultrametric input tree was obtained with BEAST v1.7.4. [81]. Statistical parsimony network analysis (SP) is commonly used to cluster haplotypes in a phylogeographical framework [82]. However, an inverse consideration of this method is proposed to allow the delimitation of coalescent populations [80]. The program TCS 1.21 [83] was used to delimitate entities on the basis of 95% statistical confidence (i.e. connection probability).

### Alignment optimization and phylogenetic tree reconstruction

Phylogenetic hypotheses were reconstructed using a concatenated dataset of three phylogenetic markers (mitochondrial 16S, COI and nuclear H3) resulting in 1210 bp. In total, 86 individuals comprising 26 carychiid morphospecies (17 *Carychium*, 9 *Zospeum*) and 38 ELs as well as three outgroup taxa (Ellobioidea: Pythiidae: *Laemodonta cubensis*; Ellobioidea: Melampodidae: *Microtralia occidentalis* and Veronicelloidea: Veronicellidae: *Veronicella cubensis*) were analyzed for the concatenated dataset (see Additional file 5). Alignment optimization was performed separately for each phylogenetic marker: 16S sequences were aligned with MAFFT 6.814 under the FFT-NS -i x 1000 algorithm implemented in the Geneious software. The initial 16S alignment had a length of 548bp. Primer sequences were deleted and the initial alignment was further modified with GBLOCKS [75] to remove ambiguously aligned internal positions and to trim the alignment at the 5’ and 3’ ends. The final 16S alignment had a length of 368 bp (67% of the initial alignment). For the nuclear H3 marker, the G-INS -i algorithm was used. Primer deletion and GBLOCKS 5’ and 3’ trimming resulted in 235 bp of the initial 330 bp (71%). The already trimmed barcoding alignment was used as the COI alignment (607 bp, 93% of initial alignment).

Topologies were estimated under three different phylogenetic reconstruction methods: Maximum Composite Likelihood (MCL), Maximum Likelihood (ML) and Bayesian inference (BI). Runs for MCL were performed in MEGA5 under the pairwise deletion option, a gamma distribution (G) with rate parameter 1 and 1,000 bootstrap replicates. RaxML 7.0.3 [84] was used to estimate the ML topology. To account for varying substitution rates between different loci and nucleotide positions, three marker-specific partitions under the GTR+G+I substitution model were set. MrModeltest 2.3 was used to distinguish between competing substitution models [85]. A thorough ML bootstrapping with 1,000 replicates was conducted. A Bayesian phylogenetic analysis was performed with MrBayes 3.2.1 [86,87] using *Veronicella cubensis* as outgroup. Three gene partitions were defined keeping the estimation of all parameters of the GTR+G+I model of evolution unlinked during the analysis. Two runs of 2,000,000 generations of the MCMC (Markov Chain Monte Carlo) were executed, sampling every 500 generations. The first 25% of the samples were discarded as burn-in to ensure sampling from the stationary phase of the model runs. The chain temperature parameter was set at 0.1. At the 2,000,000^th^ generation, the average standard deviation of split frequencies had already fell below 0.01, thus the analysis was stopped.

The concatenated alignment and the phylogenetic consensus hypothesis are deposited in TreeBASE ( http://purl.org/phylo/treebase/phylows/study/TB2:S13629).

### Alternative hypotheses testing

A model selection approach using BEAST v1.7.4 [81,88] was followed to test the monophyly of geographical closely-distributed taxa. Four taxon sets of species inhabiting a certain geographic area were created and constrained to be monophyletic, i.e. i) American *Carychium*, ii) Asian *Carychium*, iii) Dinaric *Zospeum* and iv) Alpine *Zospeum*. The monophyletic constraints were analyzed independently and compared with the results of the unconstrained topology. The Markov Chain Monte Carlo was run for 30 million generations, sampling trees and parameters every 1,000 generations. After verifying that appropriate effective sample sizes were achieved, three model selection methods were applied: a posterior simulation-based analogue of the Akaike Information Criterion (AICM) [89,90], and Bayes Factors (BF) between marginal likelihoods estimated through Path Sampling (PS) [91] and Stepping Stone Sampling (SS) [92]. We used the settings suggested on the BEAST website ( http://beast.bio.ed.ac.uk/Model_selection). Competing topological hypotheses were ranked according to the results of the AICM and the marginal likelihood values obtained with PS and SS. Differences between AIC were calculated (Δ AICM) as were Bayes Factors between competing hypotheses. A Δ>7 between AICM values of the best ranked hypothesis and the other hypotheses suggests that the latter are very unlikely [93]. A Bayes Factor_ln_ > 2.3 was considered as strong support for the hypothesis (modified guidelines of [94]).

### Estimation of relative times of divergence

The scarcity of reliable fossils of *Carychium* hinders the estimation of absolute times of divergence. *Carychium brotianum* De Loriol, 1865 from the Upper Jurassic in France is assumed to be by far the oldest carychiid fossil [95]. Our own investigations of the type material of *Carychium brotianum* deposited in the Musée Cantonal de Géologie Lausanne reject a close affiliation to Carychiidae. We rather regard the extinct lineage *Carychiopsis* Sandberger, 1872 known from the Paleocene until the Neogene or *Carychium munieri* Briart & Cornet, 1889 reported from the Early Paleocene as the oldest representatives of Carychiidae [64-66,96]. In respect to this problem, we chose to estimate only relative ages. The analysis was performed with the program BEAST v1.6.1 [81] using an uncorrelated, relaxed lognormal molecular clock model. The three genetic markers were concatenated but the parameters of the substitution and molecular clock models were independently estimated for each gene partition. The taxa *Veronicella cubensis*, *Microtralia occidentalis* and *Laemodonta cubensis* formed the outgroup taxa set. Trees were sampled from a Birth-Death tree-prior and following the GTR+G+I substitution model. The MCMC was run for 30,000,000 generations sampling trees and parameters every 1,000 generations. Effective sample size and convergence were evaluated in Tracer 1.5 [97]. The first 10% of samples were discarded as burn-in before building the maximum clade credibility (MCC) tree.

### Temporal patterns of diversification

To assess the temporal dynamics of lineage diversification, we tested several constant- and variable-rate models of diversification using a maximum likelihood approach as implemented in the R package laser [98,99]. Model-fit was evaluated using the Akaike Information Criterion (AIC) [90]. Temporal changes in the net diversification rate were evaluated by calculating the difference in the AIC score between the best fit constant-rate (pure-birth, birth-death) and variable-rate models (DDL, DDX, yule2rate). Since these comparisons are susceptible to a high Type I error rate [98], a null distribution of the test statistic was generated by calculating the ΔAIC score for 500 phylogenetic trees simulated under a pure-birth process.

### Geographic range evolution

The dispersal-extinction-cladogenesis (DEC) model implemented in Lagrange [100] was used to infer geographic range evolution. The model assumes that geographic splits for a given lineage occur along the branches of a topology rather than at bifurcation points. Changes in the geographic range of a given taxon can be accounted for by dispersal events (range expansion) or local extinction (range contraction). Alternative Lagrange analyses were run with the maximum range size set to either two or four (the maximum number of areas in the model). The patterns were largely congruent and only the results of the conservative approach are presented in which taxa are allowed a maximum range size of two, i.e. simultaneously inhabiting up to two geographic areas (here continents or mountain ranges). Given their low dispersal potential and the high proportion of continent- or cave-endemic taxa, this scenario seems more likely. The character state with the highest relative probability (fraction of the global likelihood) was plotted on the topology. In cases of similar relative probability values, all alternative scenarios were plotted to account for model uncertainty.

### Bioclimatic niche modeling

Bioclimatic niche models were constructed with the program Maxent 3.3.3 [101]. This approach is based on the principle of least assumptions. In the absence of any further information, it prefers the model with the maximum entropy. Niche parameters are extracted from occurrence points and global environmental layers and combined into taxon-specific bioclimatic envelopes. We used georeferenced data for monophyletic lineages instead of species-specific data because ambiguous species-level identifications of *Carychium* taxa are likely to produce questionable entries in public databases or museum collections. The investigation of the bioclimatic niche and macro-evolutionary changes above the species level has proven suitable in earlier studies [102]. This is understandable as traits that allow taxa to persist tend to be conserved over time [102]. The bioclimatic niches for i) the monophyletic European *Carychium* clade and ii) the monophyletic North + Central American (NC) *Carychium* clade were estimated. In total, 136 sampling points for the European clade were collected either from our own collections or the GBIF database ( http://www.gbif.org). Data from American museum collections (Carnegie Museum of Natural History, Pittsburgh; Field Museum of Natural History, Chicago; Florida Museum of Natural History, Gainesville) and entries within the GBIF database are used as distribution data for the NC-clade resulting in 241 sampling points for this model. All 19 bioclimatic variables of the WorldClim project [103] were used in highest resolution (~30 arc-seconds) in order to discover potential, fine-scale patterns affecting the distribution of carychiid microgastropods. Almost all included georeference points (>98%) were accurate to about less than 1 km, which justifies the implementation of bioclimatic variables in their highest resolution. We performed five cross-validated Maxent runs and considered grid cells with a cumulative probability of more than 10 (from a range of 0–100) as suitable [104,105]. The area under the ROC curve (AUC) gave an evaluation of the projections’ overall quality. An AUC score above 0.7 is considered good model performance [106]. Outputs are generalized clade-based models. Such a model design can slightly overestimate taxon-specific bioclimatic envelopes and can lead to false-positive model results (i.e. predicted habitat suitability but biological absence of taxa).

### Niche similarity

ENMtools [107] was used for the comparative analysis of environmental niche models of the European and NC-clade. Niche overlap between the two clades was assessed using three different similarity statistics: Schoener's D [108], the I statistic [109], and the relative rank test [110]. Schoener's D and I calculate the difference in standardized suitability scores for each grid cell. The relative rank test provides an estimate of the congruence of relative ranks of suitability for each grid cell. All three measurements can have a range between 0 (non-overlapping niches) and 1 (identical niches). To calculate niche similarity, climatic niche models were cross-projected onto the geographic region of the other clade.

## Competing interests

The authors declare that they have no competing interests.

## Authors’ contributions

AMW, JS, AJ and AKK designed the study. AJ, AMW and RS collected the specimens. AWM produced the sequences and together with JS acquired the biostatistical data. AMW, JS and EZ conducted the biostatistical analyses. AMW was responsible for the first draft of the manuscript. All authors gave conceptual comments, read and approved the final manuscript.

## Supplementary Material

Additional file 1 Figure *.tif. Schematic visualization for the constrained model selection approaches. The unconstrained phylogenetic hypothesis was tested against four competing evolutionary scenarios in a model selection approach (refer to Table 3). Both scenarios concerning *Carychium* microgastropods are depicted, since the constraints likewise affected the splitting order of ancient nodes. E = Europe; A = Asia; N+C = North+Central America excl. *C. nannodes*; N = *C. nannodes*. Black dots indicate posterior probability ≥ 0.98. A: phylogenetic unconstrained hypothesis. A_1a_ = *C.* cf. *pessimum*; A_1b_ = *C. nipponense*; A_2_ = *Carychium* sp.3; A_3_ = *C.* cf. *noduliferum*. B: monophyletic Asian *Carychium*. C: monophyletic American *Carychium*. A_1_ = *C.* cf. *pessimum* + *C. nipponense*; A_2_ = *C.* cf. *noduliferum* + *Carychium* sp.3. Click here for file

Additional file 2 Figure *.tif. Bioclimatic niche models. The suitable bioclimatic conditions of the native monophyletic North + Central American (A; red) and European (B; yellow) clades are illustrated. Niche projections for these clades and between the areas are depicted in C and D. Black triangles indicated occurrence data of native (in A and B) and schematic snails of introduced taxa (in C). A: Potential distribution of the native North + Central American clade. B: Potential distribution of the native European clade. C: Projected distribution of the European clade in North + Central America. Non-native European *Carychium minimum* (CM) and *C. tridentatum* (CT) populations are indicated. D: Projected distribution of the North + Central American clade in Europe. Click here for file

Additional file 3 Figure *.tif. Distribution of bio2 (diurnal range) in North and Central America (TIFF 807 kb). North and Central American diurnal range values have a minimum of 1.8 (black) and a maximum of 21.4 (white) degrees Celsius (°C). Click here for file

Additional file 4 Table *.xlsx. Non-native *Carychium* populations in North America. Locality information and literature sources for non-native *Carychium* populations (based on morphospecies IDs) in North America are listed. Specimens from localities indicated by an asterisk (*) are validated by DNA barcoding as the respective *Carychium* species [111-116]. Click here for file

Additional file 5 Table *.xlsx. BOLD barcode identifier (COI) and NCBI accession numbers (16S and H3). EL = evolutionary lineage; # = specimen number. ^1^ type locality population, regarded as *C. costaricanum*. ^2^ cave population from region mentioned in phenotype description of *Z. isselianum*. ^3^ type locality population of *Z. spelaeum schmidti*. ^4^ cave population of *Z. suarezi* from cave locality mentioned in phenotype description, ^5^ H3 sequence of #152 upon request (too short for GenBank deposition). Click here for file
