# Supplementary figures and images for: Evolution of microgastropods (Ellobioidea, Carychiidae): integrating taxonomic, phylogenetic and evolutionary hypotheses
Source: BMC Evol Biol. 2013 Jan 23;13:18. doi: 10.1186/1471-2148-13-18 (PMC3558328; doi:10.1186/1471-2148-13-18)

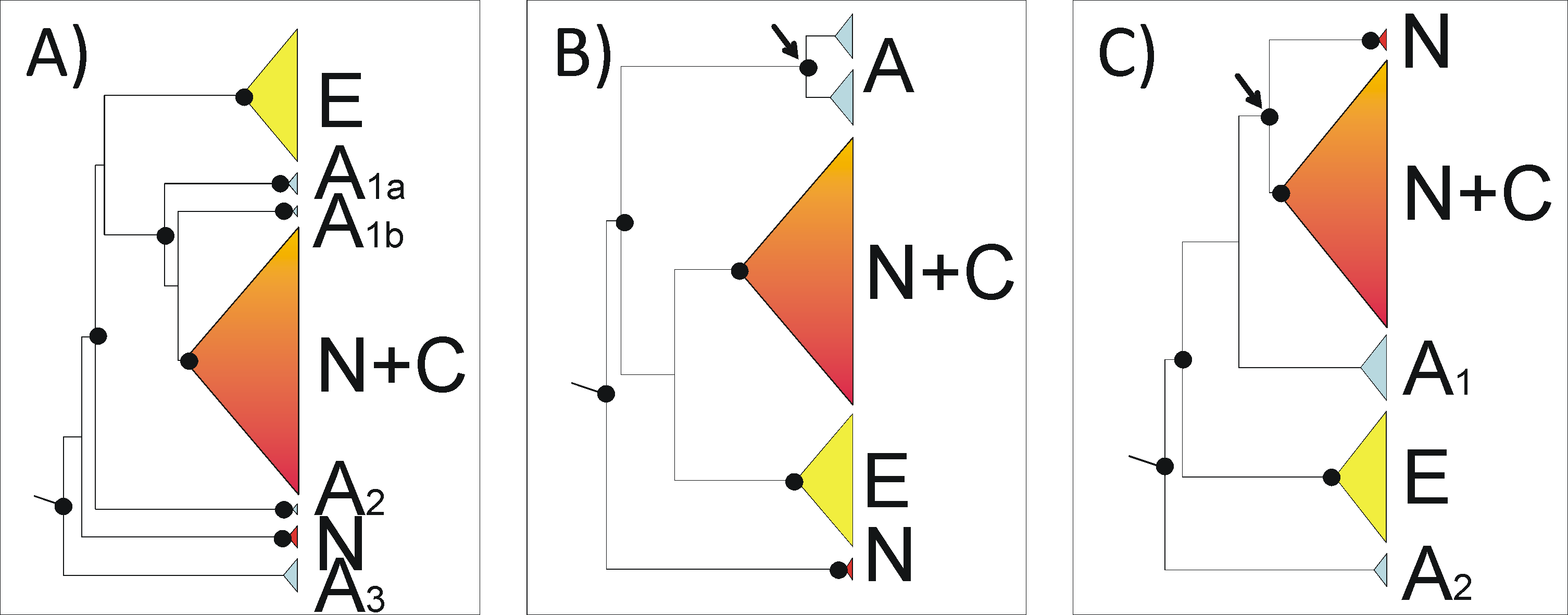

Supplement: Additional file 1 — Figure *.tif. Schematic visualization for the constrained model selection approaches. The unconstrained phylogenetic hypothesis was tested against four competing evolutionary scenarios in a model selection approach (refer to Table 3). Both scenarios concerning Carychium microgastropods are depicted, since the constraints likewise affected the splitting order of ancient nodes. E = Europe; A = Asia; N+C = North+Central America excl. C. nannodes; N = C. nannodes. Black dots indicate posterior probability ≥ 0.98. A: phylogenetic unconstrained hypothesis. A1a = C. cf. pessimum; A1b = C. nipponense; A2 = Carychium sp.3; A3 = C. cf. noduliferum. B: monophyletic Asian Carychium. C: monophyletic American Carychium. A1 = C. cf. pessimum + C. nipponense; A2 = C. cf. noduliferum + Carychium sp.3. [file 1471-2148-13-18-S1.tiff]

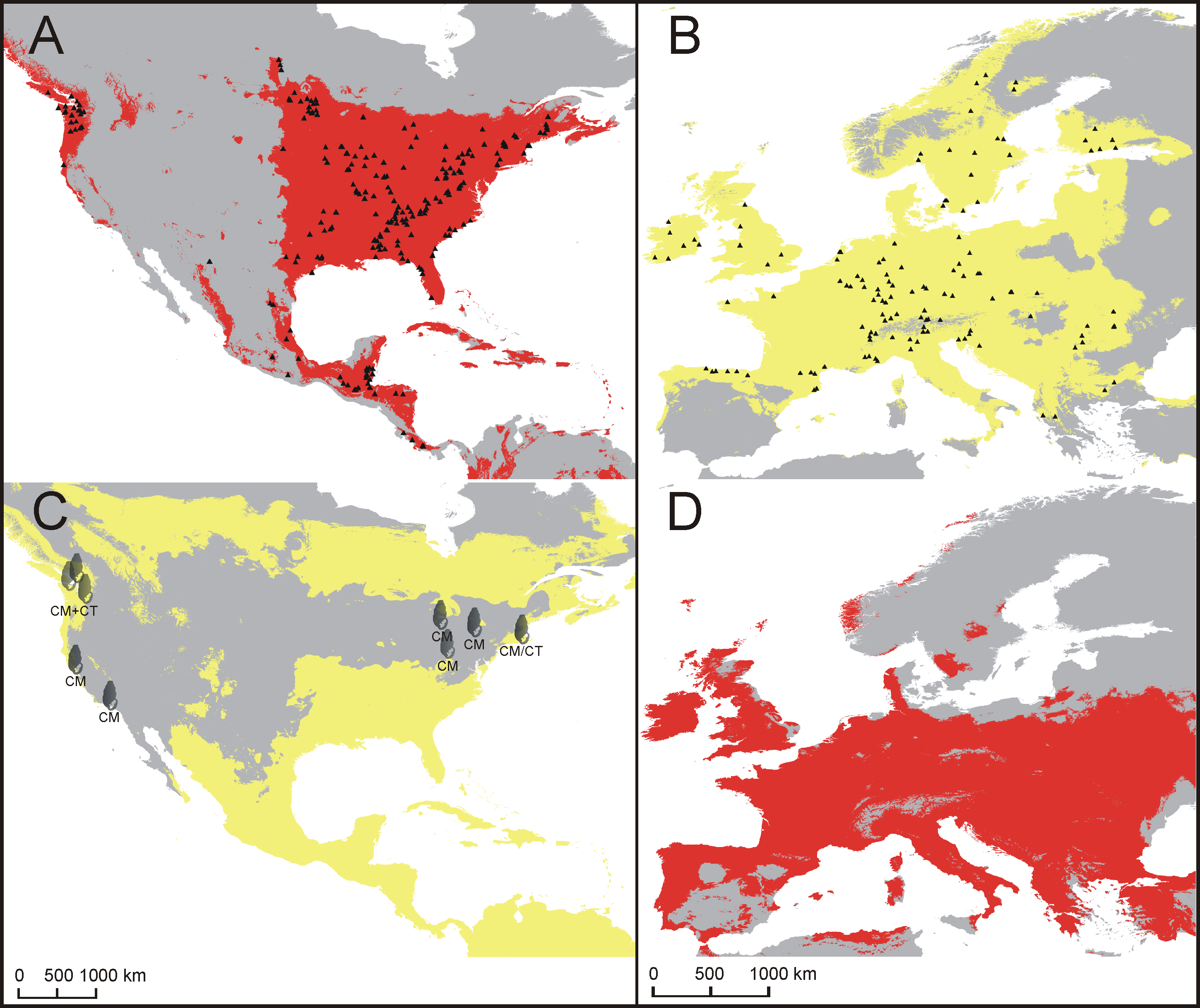

Supplement: Additional file 2 — Figure *.tif. Bioclimatic niche models. The suitable bioclimatic conditions of the native monophyletic North + Central American (A; red) and European (B; yellow) clades are illustrated. Niche projections for these clades and between the areas are depicted in C and D. Black triangles indicated occurrence data of native (in A and B) and schematic snails of introduced taxa (in C). A: Potential distribution of the native North + Central American clade. B: Potential distribution of the native European clade. C: Projected distribution of the European clade in North + Central America. Non-native European Carychium minimum (CM) and C. tridentatum (CT) populations are indicated. D: Projected distribution of the North + Central American clade in Europe. [file 1471-2148-13-18-S2.tiff]

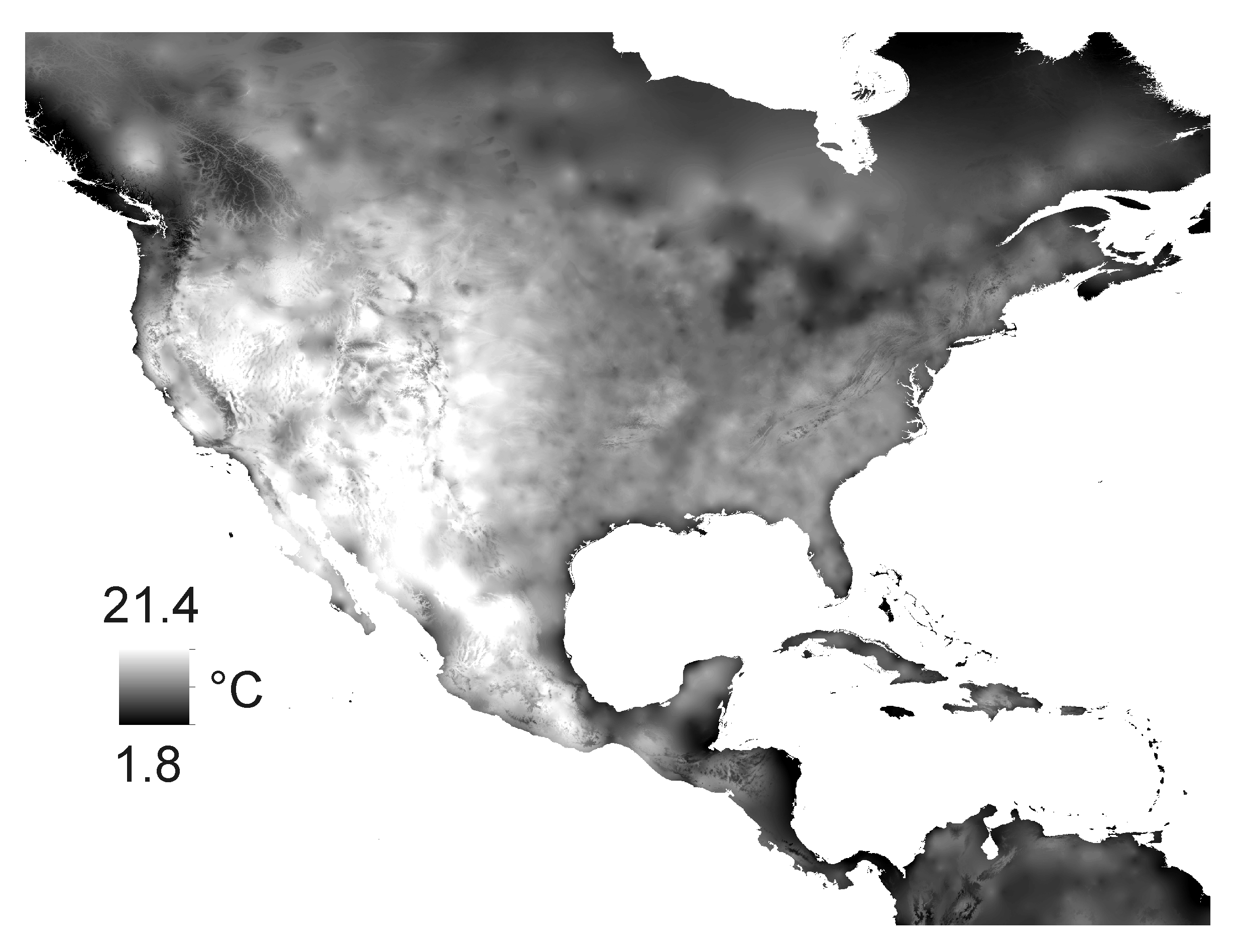

Supplement: Additional file 3 — Figure *.tif. Distribution of bio2 (diurnal range) in North and Central America (TIFF 807 kb). North and Central American diurnal range values have a minimum of 1.8 (black) and a maximum of 21.4 (white) degrees Celsius (°C). [file 1471-2148-13-18-S3.tiff]
